# X-ray irradiation triggers immune response in human T-lymphocytes via store-operated Ca^2+^ entry and NFAT activation

**DOI:** 10.1085/jgp.202112865

**Published:** 2022-04-13

**Authors:** Dominique Tandl, Tim Sponagel, Dalia Alansary, Sebastian Fuck, Timo Smit, Stephanie Hehlgans, Burkhard Jakob, Claudia Fournier, Barbara A. Niemeyer, Franz Rödel, Bastian Roth, Anna Moroni, Gerhard Thiel

**Affiliations:** 1 Department of Biology, Technische Universität Darmstadt, Darmstadt, Germany; 2 Molecular Biophysics, University of Saarland, Center for Integrative Physiology and Molecular Medicine, Homburg/Saar, Germany; 3 Department of Radiotherapy and Oncology, Goethe-University, Frankfurt am Main, Germany; 4 Department of Biophysics, GSI Helmholtzzentrum für Schwerionenforschung, Darmstadt, Germany; 5 Department of Biosciences and CNR IBF-Mi, Università degli Studi di Milano, Milano, Italy

## Abstract

Radiation therapy efficiently eliminates cancer cells and reduces tumor growth. To understand collateral agonistic and antagonistic effects of this treatment on the immune system, we examined the impact of x-ray irradiation on human T cells. We find that, in a major population of leukemic Jurkat T cells and peripheral blood mononuclear cells, clinically relevant radiation doses trigger delayed oscillations of the cytosolic Ca^2+^ concentration. They are generated by store-operated Ca^2+^ entry (SOCE) following x-ray–induced clustering of Orai1 and STIM1 and formation of a Ca^2+^ release–activated Ca^2+^ (CRAC) channel. A consequence of the x-ray–triggered Ca^2+^ signaling cascade is translocation of the transcription factor nuclear factor of activated T cells (NFAT) from the cytosol into the nucleus, where it elicits the expression of genes required for immune activation. The data imply activation of blood immune cells by ionizing irradiation, with consequences for toxicity and therapeutic effects of radiation therapy.

## Teaser

The authors expose T lymphocytes to x-ray doses relevant in tumor therapy. This triggers an immune response via activation of store-operated Ca^2+^ entry.

## Introduction

Ionizing radiation (IR) is a universal tool in medical diagnostics and therapy. At higher doses (>1 Gy), it serves as a major component in antitumor treatment (Delaney et al., 2005). A major dose limitation of radiation therapy is posed by toxic effects on the surrounding heathy tissue. In that context, peripheral blood mononuclear cells (PBMCs) cover an interesting population of cells. They are sensitive to IR (Bauer et al., 2011; Heylmann et al., 2014) and, while cycling through the bloodstream of the irradiated tissue, are unavoidably exposed. Further, high-dose exposure results in a suppression of immune functions (Stoecklein et al., 2015). This includes, among others, killing of blood cells (Donnelly et al., 2010), induction of cell cycle arrest of immune cells (Goans and Waselenko, 2005), but also triggering of proinflammatory processes (Di Maggio et al., 2015; Lumniczky et al., 2017). Furthermore, recent findings indicate an immune-stimulatory effect of high-dose radiation, with several studies describing a synergistic impact on local and distant tumor control, in particular when radiation therapy is combined with anticheckpoint programmed death 1 (PD-1) receptor or its ligand (PD-L1) immunotherapy (Frey et al., 2017; Sharabi et al., 2015). Finally, recent data suggest that irradiation at lower doses displays anti-inflammatory or immune-modulatory effects, with consequences for immune surveillance of noncancerous cells (Cuttler, 2020).

A previous study reported that x-ray irradiation at a low to medium dose elicits cellular responses that are typically associated with immune stimulation in naive T-lymphocytes (Voos et al., 2018). These include an increase in cell diameter, up-regulation of CD25 membrane expression, IL-2, and INF-γ synthesis, enhanced integrin-mediated adhesion of Jurkat cells, as a model for T cells, or PBMCs to endothelial cells. Since many of the aforementioned mechanisms are mediated by a Ca^2+^ signaling cascade, we anticipate a causal interrelation between radiation stress and induction of intracellular Ca^2+^ signaling cascades (Heylmann et al., 2014; Voos et al., 2018). Here, we aimed to examine whether clinically relevant x-ray doses between 0.5 and 5 Gy trigger Ca^2+^ signaling events in T cells and whether these signal transduction cascades are relevant for typical immune-stimulating processes. We observed that x-ray irradiation triggered in Jurkat cells a depletion of intracellular Ca^2+^ stores, followed by long-lasting episodes of Ca^2+^_cyt_ oscillations after a delay of some 10 min. These oscillations are mediated by store-operated Ca^2+^ entry (SOCE) via a radiation-induced plasma membrane (PM) clustering of calcium release–activated calcium modulator 1 and the stromal interaction molecule 1 (Orai1/STIM1). As in the case of immune stimulation by antigens, the x-ray–induced formation of calcium release–activated calcium (CRAC) channels in turn mediates the nuclear translocation of the transcription factor nuclear factor of activated T cells (NFAT). This stimulus-induced and Ca^2+^-dependent nuclear import is a well-known essential step for cytokine production, proliferation, and immune competence (Vaeth and Feske, 2018) and can therefore be used as a criterion for an activation of T cells.

## Materials and methods

### Cell culture

Jurkat cells (ACC 282) were purchased from the German Collection of Microorganisms and Cell Cultures. They were grown in RPMI 1640 medium (Thermo Fisher Scientific), supplemented with 10% heat-inactivated FCS (PAA) and 50 U/ml penicillin plus 5 µg/ml streptomycin (Sigma-Aldrich). PBMCs were isolated from blood of healthy volunteers using density-gradient centrifugation (Biocoll Separating Solution; Biochrom) and maintained in RPMI 1640 medium with 10% FCS, 50 U/ml penicillin and 5 µg/ml streptomycin before assays as described previously (Voos et al., 2018).

### Generation of CRISPR-Cas9–mediated Orai1 knockout Jurkat cells

Sequence-specific gRNA for 5′-UTR Orai1 (5′-GTG​AGG​CCG​GGC​CCG​CGT​AGG​GG-3′) was designed using an online tool (http://crispr.dbcls.jp/) and subcloned into pX459 (pSpCas9(BB)-2A-Puro V2.0; Addgene plasmid 62988) using the BbsI recognition site. Jurkat E6.1 T cells were transfected with the generated CRISPR plasmids using Nucleofector 4D electroporation kit SF (Lonza) according to the manufacturer’s instructions. Cells were then cultured for 72 h after transfection in medium containing 1 µg/μl puromycin to select transfected cells. A monoclonal cell line was generated by infinite dilution, and the efficiency of protein deletion was evaluated by Western blot analysis. Immunoblots were probed with anti-Orai1 (O8264; Sigma-Aldrich).

### Cell irradiation and treatments

Cells were exposed to x-ray irradiation in T_35_ Petri dishes using an Isovolt 160 Titan E source with a voltage of 90 kV and 33.7 mA (GE Sensing & Inspection Technologies) with a dose rate of 0.055 Gy/s. Ionomycin (ab120370; Abcam), thapsigargin (Tg; T9033; Sigma-Aldrich), and the cell-permeable Ca^2+^ sensors Fluo-4 AM (F14201), Fura-2 AM (F1221), and Mag-Fluo-4 (M14206; all Thermo Fisher Scientific) were dissolved in DMSO and added to external solution immediately before experiments, with final concentrations mentioned in text. To activate human T cells, ImmunoCult Human CD3/CD28/CD2 T cell activator (short T-Ac; 10990; Stemcell Technologies) was added to the cell culture medium (25 μl per 1 ml cell suspension). The cell-permeable organelle trackers Mito-Tracker Green FM (M7514) and ER-Tracker Red (E34250), nucleus-staining Hoechst dye (H1399), and PM tracker CellMaskOrange (C10045; Thermo Fisher Scientific) were used according to the manufacturer’s recommendations, diluted in external microscopy buffer, for 30 or 10 min, respectively, at 37°C. Subsequently, cells were washed and resuspended in dye-free microscopy buffer. CRAC channels were blocked by inhibitors Synta66 (SML1949; Sigma-Aldrich) and Pyr6 (203891; Sigma-Aldrich) dissolved in DMSO and resuspended in medium or microscopic solution with final concentrations of 10 or 5 µM, respectively.

### Determination of cell diameters

Jurkat cell diameters were measured with an EVE automatic cell counter (NanoEnTek) and corrected manually using EVE PC.LNK 1.0.3 software. Cell viability was determined by using trypan blue exclusion.

### Confocal laser scanning microscopy

Confocal laser scanning microscopy was performed on a Leica TCS SP or SP5 II system (Leica Microsystems) equipped with a 40× 1.30 oil UV (HCX PL APO), a 63× 1.4 oil UV (HCX PL APO lambda blue), or a 100× 1.44 oil UV objective (HCX PL APO CS). The external buffer used for microscopy contained (in mM) 140 NaCl, 4 KCl, 1 MgCl_2_, 5 Mannitol, 10 HEPES, and 2 CaCl_2_, pH 7.4, with osmolarity of 310 mosmol/l. Live-cell imaging of changes in Ca^2+^ and translocation of NFAT-GFP were performed as described in Voos et al. (2018) and Kehlenbach et al. (1998), respectively. STIM1-eYFP and ORAI-eCFP were transiently expressed as described in Zheng et al. (2018). To enable a gentle adhesion of the cells to the glass coverslips (Ø 25 mm), they were prepared by cleaning in a plasma furnace (Zepto-B; Diener Electronic) and coated with one layer of PBS/5% BSA in a spincoater (PIN150; SPS Europe Spincoating) and a second layer of 0.01% poly-L-lysine (molecular weight 75–150 kD).

For monitoring Ca^2+^, Jurkat cells were loaded with the cell-permeable Ca^2+^ sensor Fluo-4-AM, Mag-Fluo-4, or Fura-2-AM. For recordings of Ca^2+^_cyt_ with Fluo-4 and Ca^2+^ of intracellular Ca^2+^ stores with Mag-Fluo-4, which is suitable for monitoring depletion of Ca^2+^ from ER (Rossi and Taylor, 2020), cells were incubated for 30 min in microscopy buffer at a final concentration of 1 µM. The calcium dye was subsequently removed by washing cells with dye-free buffer. Calcium signals were recorded for a time interval of 5 s for 60–240 min in total, with an image resolution of 1,024 × 1,024 pixels and a scan speed of 400 Hz. Transfection of the immune cells for transiently expressed proteins was accomplished with lipofectamine 2000 (11668019; Thermo Fisher Scientific) according to the manufacturer’s instructions. Live-cell analysis of heterologously expressed NFATc2-GFP and STIM1-eYFP/Orai1-eCFP localization was performed using the confocal laser-scanning microscopes mentioned above. The microscopy settings were as follows: image resolution 1,024 × 1,024 pixels, scan speed 200 Hz, and time interval 30 s for 30–100 min in total.

For measurements with Fura-2, Jurkat cells were loaded with 1 µM Fura-2-AM in medium on a rocking shaker at room temperature for 20–25 min and seeded on glass coverslips coated with poly-ornithine (0.1 mg/ml) and allowed to settle for 5 min. Coverslips were assembled into a self-built perfusion chamber with small volume and high solution exchange rate at room temperature. The external Ca^2+^ Ringer solution contained (in mM): 145 NaCl, 2 MgCl_2_, 4 KCl, 10 Glucose, 10 HEPES, and 0.5 CaCl_2_ (0.5 Ca^2+^ Ringer) or no CaCl_2_ but 1 EGTA (0 Ca^2+^ Ringer; pH 7.4 with NaOH). Images were acquired at 0.2 Hz and analyzed with TILLVision followed by Igor software.

### FRET analysis

FRET experiments with Jurkat cells transiently expressing STIM1-eYFP/Orai1-eCFP were examined with a Leica TCS SP5 II confocal microscope. Filters were set with CFP (458 excitation/460–490 emission), YFP (514 excitation/530–550 emission), and FRETraw (458 excitation/530–550 emission). Live-cell images were obtained every 30 s at room temperature with a 100× 1.44 oil UV objective (HCX PL APO CS) for a time period of 30 min. Three-channel corrected FRET was calculated based on the following equation: FRETc = Fraw − *Fd*/*Dd* · FCFP − *Fa*/*Da* · FYFP, where FRETc represents the corrected total amount of energy transfer; Fraw is the measured FRET signal; *Fd*/*Dd* is the measured bleed-through of eCFP via YFP filter (0.473); and *Fa*/*Da* represents measured bleed-through of YFP through CFP filter (0.049). To reduce variations caused by differences in expression levels of CFP, the FRETc values were normalized to value of donor fluorescence (FCFP). To minimize the effect of variations of YFP expression levels on FCFP-normalized FRET signals (FRETN), and to show the relative changes compared with resting levels, figures are shown as ΔFRETN/FRETNrest.

### Immunofluorescence staining

Jurkat cells were fixed on BSA/poly-L-lysine–coated glass coverslips 15, 30, 45, 60, or 90 min after treatment using 4% paraformaldehyde and stained with primary antibodies for STIM1 (PA1-46217; Thermo Fisher Scientific), Orai1 (NBP1-75523; Novus Biologicals, or O8264; Sigma-Aldrich), and NFATc2 (MA1-025; Thermo Fisher Scientific). Antibodies were applied at a 1:200 dilution in PBS, and coverslips were shaken overnight at 4°C. Next, cells were washed and incubated with anti-rabbit IgG Alexa Fluor 488 (Alx488) secondary antibody (A32731; Thermo Fisher Scientific), anti-mouse IgG Alx488 secondary antibody (A32723; Thermo Fisher Scientific), or anti-mouse IgG Alx647 secondary antibody (A32728; Thermo Fisher Scientific).

Formation of Stim1/Orai1 clusters in the PM were detected and quantified as follows: fluorescent images of Jurkat cells immunostained with Alx647 (magenta Orai1) and Alx488 (green STIM1) were merged in Fiji software. To avoid unspecific localization of the Alx488 signal in the PM, the global signal intensity was reduced to 5% of the mean Alx488 fluorescence in the cytoplasm, with the effect that only pixels with a high fluorescence intensity remained. The remaining colocalization of Alx647 and Alx488 pixels were classified as STIM1/Orai1 clusters when colocalized pixels in direct contact with these settings covered an area with a diameter between 0.3 and 1 µm. Cells that exhibited five or more of such clusters were classified as STIM1/Orai1 positive.

### Statistics

Data are expressed as mean ± SD or SEM of three or more independent experiments; number of biological replicates (*n*) or independent experiments (*N*) are given in the figures and/or text. Significance was estimated by unpaired Student’s *t* test.

### Online supplemental material

**Figure S1.**
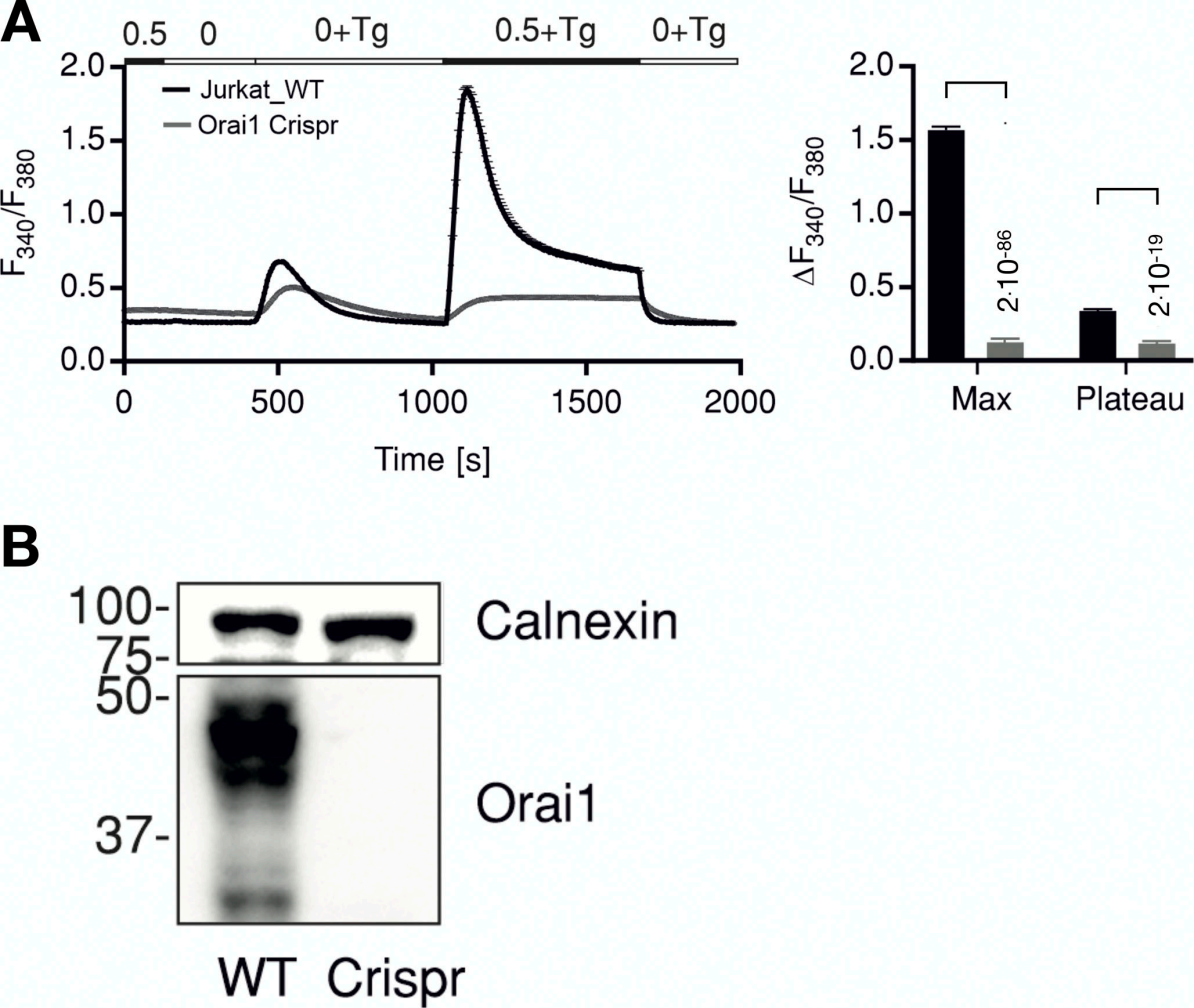
**Knockout of Orai1 in Jurkat cells abolishes Tg induced Ca**^**2+**^ **release. (A)** Average traces showing changes in Ca^2+^_cyt_ as indicated by fluorescence ratio (F_340_/F_380_) of ratiometric calcium dye Fura-2 over time in response to Tg-induced ER calcium store depletion and Ca^2+^ readdition by changing extracellular solutions as shown in the bar above. Measurements were done in control Jurkat cells (WT, black) or in cells where Orai1 was deleted (Orai1-Crispr, gray). The right panel shows quantification of maximum (Max) and steady-state (Plateau) changes in fluorescence ratio + SD from 135 to 176 cells measured in *N* = 3 independent experiments. Statistical differences between WT and Crispr cells were analyzed by unpaired Student’s *t* test, and respective P values are given in the figure. **(B)** Western blot analysis of cells measured in A showing deletion of Orai1 in Crispr-Cas9–treated cells. Source data are available for this figure: SourceData FS1.

**Figure S2.**
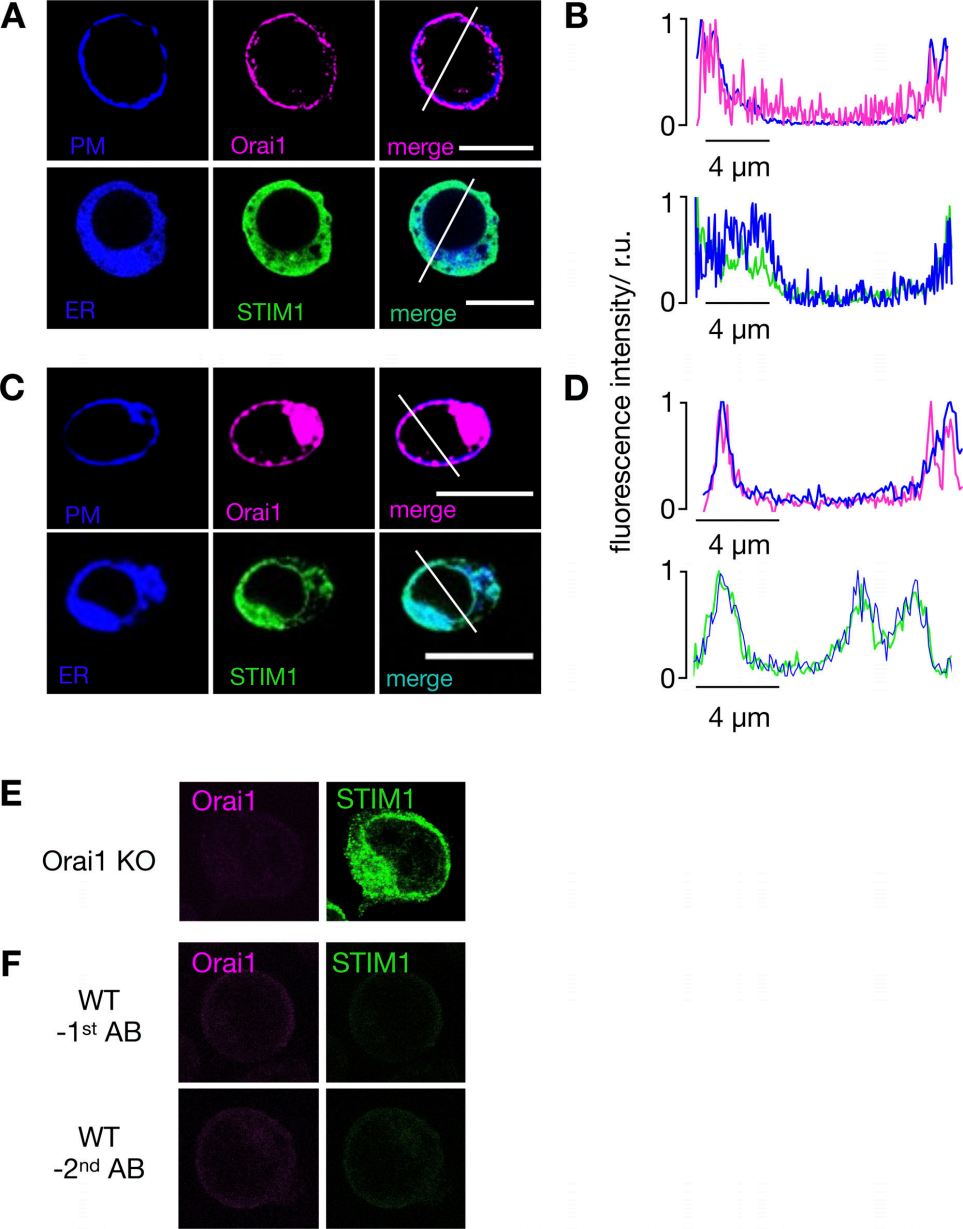
**In resting Jurkat cells, STIM1 and Orai1 are located in the ER and PM, respectively. (A and C)** Confocal images of cellular distribution of endogenous STIM1 and Orai1 in Jurkat cells (A) and PBMCs (C). PM and ER of cells (first row) were stained with CellMaskOrange and ER-tracker red, respectively. Images shown as false color in blue. The second row shows immunostaining of STIM1 (green) and Orai1 (magenta) and secondary antibody tagged with Alx488 and Alx647, respectively. An overlay of both channels is shown in right column. **(B and D)** Line plots for each marker were taken in positions report in merged images. Fluorescence intensity of either Orai1/PM (B) or STIM1/ER (D) were normalized to the highest value of each signal; the colors of line plots correspond to those in images. All scale bars, 10 μm. The antibodies for detecting STIM1 and Orai1 are specific. **(E)** Jurkat cells in which Orai1 was knocked out (Fig. S2) generate no more signal in immunostaining with Orai1 antibody, while still producing signal with STIM1 antibody. **(F)** Staining of WT Jurkat cells in which either the primary STIM1 or Orai1 antibodies (top row) or the respective secondary antibodies (bottom row) were left out generated no appreciable fluorescent signals.

Fig. S1 shows that under resting conditions, Jurkat cells exhibit STIM1 and Orai1 in the ER and PM, respectively. Additional experiments confirm that the antibodies used in this study are specific for STIM1 and Orai1 in Jurkat cells. Fig. S2 shows that a knockout of Orai1 in Jurkat cells abolishes Tg-induced Ca^2+^ release. Video 1 illustrates the dynamics of x-ray–induced oscillation of Ca^2+^_cyt_ in Jurkat cells. Video 2 presents a Jurkat cell in which, after x-ray exposure, NFAT is translocating from cytosol to nucleus.

## Results

### Ionizing irradiation elicits a delayed Ca^2+^ response in Jurkat cells

**Figure 1.**
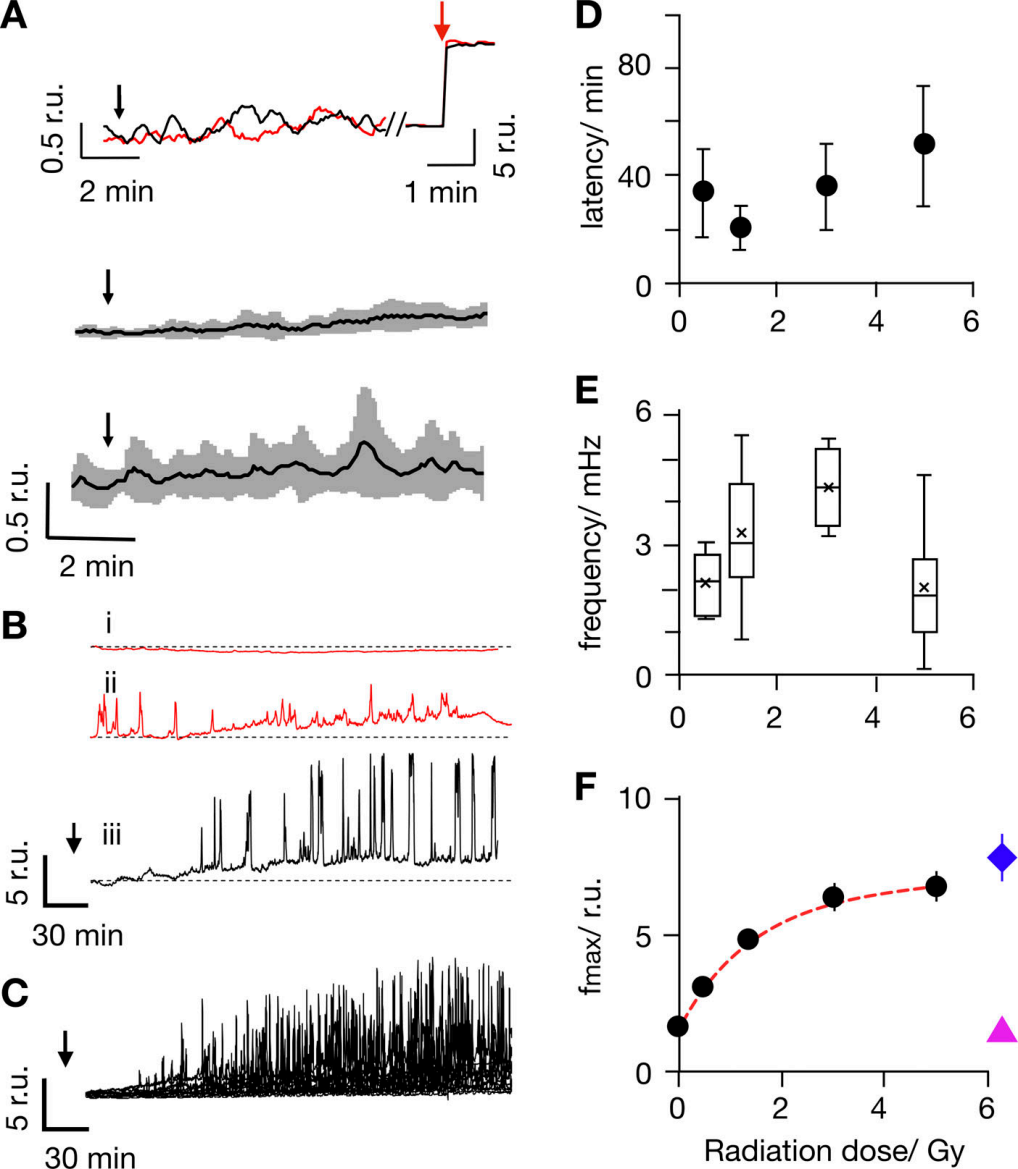
**Ionizing irradiation elicits delayed Ca**^**2+**^_**cyt**_ **oscillations with distinct frequencies and amplitudes. (A)** Representative Fluo-4 signals report constant Ca^2+^_cyt_ in individual Jurkat cells (top). Fluorescence was recorded in real time before, during, and after irradiation with 1 Gy (red) and 10 Gy (black) x rays at times indicated by black arrows. The same cells responded with a maximal fluorescence increase after addition of 1 µM ionomycin (red arrow). Mean Fluo-4 intensity from cells irradiated at arrow with 1 Gy (middle) or 10 Gy (bottom) x rays. Data are means (black) ± SD (gray) from experiments as in A, with 15 cells for each dose. **(B)** Representative long-term measurements of Fluo-4 intensity in Jurkat cells, of which two were not irradiated (i, ii, ctrl., red) and the other was exposed to 5 Gy x ray (iii, black). Time of x-ray exposure is indicated by black arrows. While unirradiated control cells maintain a stable Fluo-4 signal (i) or reveal irregular oscillations (ii), the irradiated cell starts to oscillate after a lag time (iii). **(C)** Overlay of Fluo-4 signal from 15 individual cells after irradiation with 5 Gy; time of x-ray exposure is indicated by black arrow. **(D–F)** Latency time between onset of Ca^2+^_cyt_ oscillations after irradiation, oscillation frequency (E), and maximal amplitude of oscillation (F) as a function of irradiation dose. The colored symbols show different levels of Fluo-4 fluorescence intensity: Fluo-4 intensity elicited by 5 Gy in Ca^2+^ free external buffer including 5 mM EGTA (magenta triangle) and maximal Fluo-4 intensity after adding 1 µM ionomycin (blue diamond). Data are mean values ± SD from ≥25 cells per dose; box plot indicates 25th and 75th percentiles of data; and whiskers indicate 5% and 95% limits. All Ca^2+^_cyt_ measurements in response to x-ray irradiation were performed in buffer containing 2 mM Ca^2+^.

It was previously shown that x-ray irradiation triggers partial immune stimulation in T cells, which is mediated by an oscillatory increase in the concentration of free Ca^2+^ in the cytoplasm (Ca^2+^_cyt_; Voos et al., 2018). To unravel a causal relationship between IR and Ca^2+^_cyt_ signaling, we monitored the immediate impact of x-ray exposure on the level of this second messenger in Jurkat cells. Individual cells loaded with the fluorescent Ca^2+^_cyt_ dye Fluo-4 were imaged in real time with a fluorescence microscope directly coupled to an x-ray source. The exemplary data in Fig. 1 A, top, from two cells exposed to x-ray doses of 1 and 10 Gy show that this type of irradiation was not eliciting any appreciable increase in Ca^2+^_cyt_ during or 10 min after irradiation. This absence of a Ca^2+^_cyt_ response to both doses of irradiation was confirmed by repeating the same experiments with additional cells. The mean data in Fig. 1 A show that neither 1 Gy (Fig. 1 A, middle) nor 10 Gy (Fig. 1 A, bottom) of x rays elicited any appreciable early Ca^2+^_cyt_ responses to irradiation beyond the scatter of the data. Combined with the finding that Ca^2+^_cyt_ can be elevated in the same cells by ionomycin (Fig. 1 A, top), these data suggest that IR exposure has no immediate impact on Ca^2+^_cyt_ in Jurkat cells; the data also underpin that x-ray irradiation causes no unspecific leakage of cell membranes.

**Figure 2.**
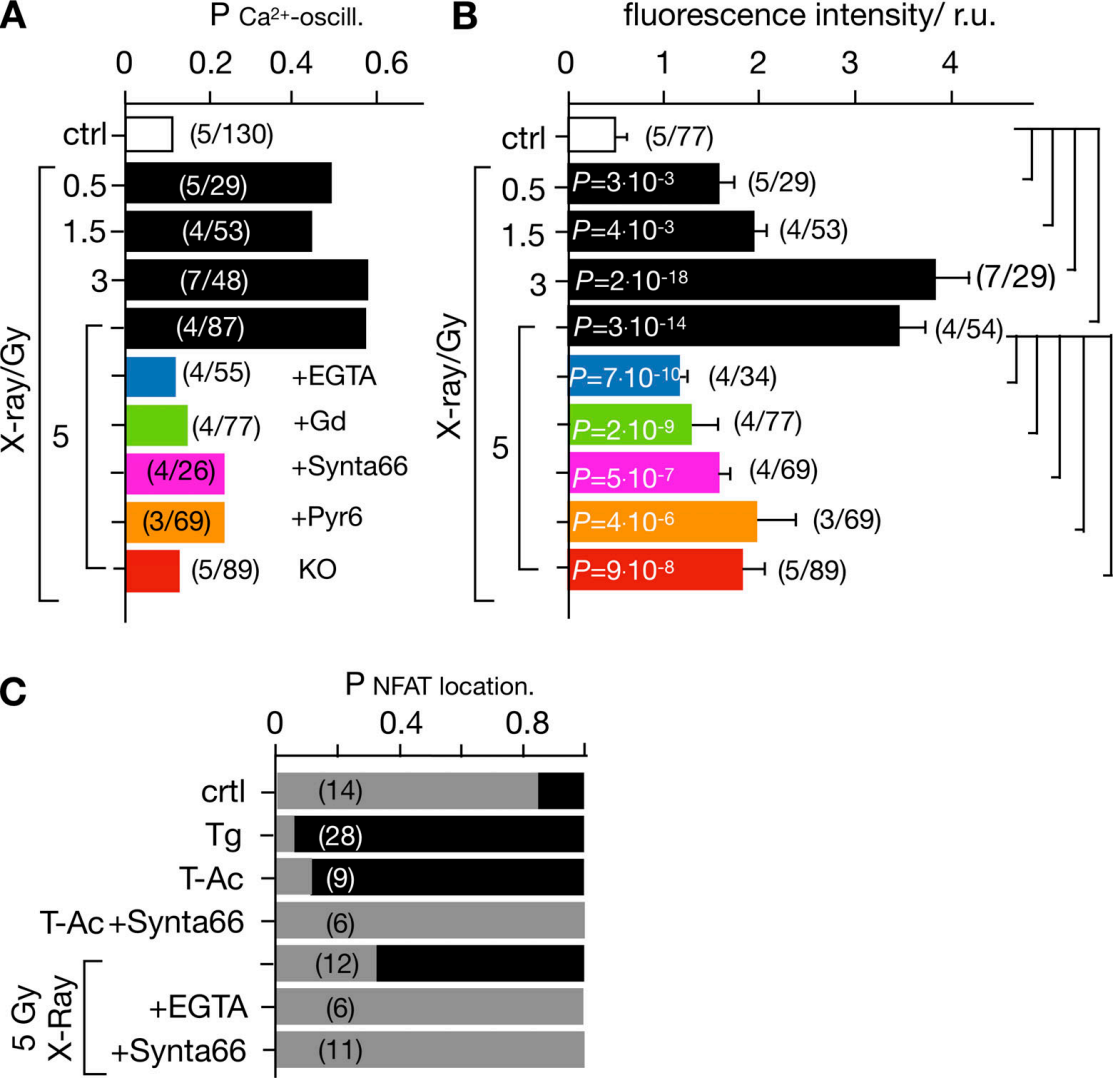
**Radiation-induced Ca**^**2+**^_**cyt**_ **oscillations and nuclear NFAT translocation are triggered by x** **rays and abolished after inhibition of Ca**^**2+**^ **influx.** The probability for detecting Ca^2+^_cyt_ oscillations (P_Ca_^2+^_-oscill*.*_) in Jurkat cells as in Fig. 1 Biii. A population of cells was imaged under the aspect of finding ≥10 min after start of imaging Ca^2+^_cyt_ oscillations defined as ≥5 repetitive Ca^2+^_cyt_ spikes. Cells were either not irradiated (ctrl, open bar) or exposed to x-ray doses of 0.5–5 Gy in the absence (black bars) or presence of 5 mM EGTA (blue bar), 5 μM Gd^3+^ (green bar), 10/5 µM CRAC channel inhibitor Synta66 (magenta), or Pyr6 (orange). The same experiments were also performed with cells in which Orai1 was knocked out (KO; red bar). **(B)** Mean fluorescence (± SD) collected over a time window of 60–120 min after start of imaging from cells treated as in A. **(C)** Probability of detecting NFAT in cytosol (gray bar) or in nucleus (black bar) as in Fig. 6 A in untreated/nonirradiated control cells (crtl) and cells treated with 2 µM Tg or with 25 μl/ml ImmunoCult Human CD3/CD28/CD2 T-Ac without and with 10 µM Synta66. Other cells were exposed to 5 Gy x rays without or with 5 mM EGTA or 10 µM Synta66. Numbers in brackets in A–C give number of experiments (*N*)/total number of cells analyzed (*n*). Statistical differences between treatments in B analyzed by unpaired Student’s *t* test, with respective P values given in the figure.

To capture potentially delayed Ca^2+^_cyt_ responses, Fluo-4 fluorescence was monitored over an extended time window. Representative recordings in Fig. 1 B indicate that most untreated cells maintained a constant low Ca^2+^_cyt_ over 3 h of recording (Fig 1 Bi). In 130 control cells, we observed in only 20% of the cells some spontaneous and nonperiodic excursions in Ca^2+^_cyt_; the latter were mostly already visible at the start of the imaging (Fig. 1 Bii). In 89 of the cells exposed to 5 Gy, >50% exhibited characteristic delayed Ca^2+^_cyt_ oscillations. In the representative example in Fig. 1 Biii, the cell started oscillating after a lag time of 65 min (Video 1). Similar delayed and long-lasting Ca^2+^_cyt_ oscillations were observed in ≥50% of the cells irradiated with 5 Gy (Fig. 1 C). The remaining irradiated cells either maintained a constant Ca^2+^_cyt_ or showed unspecific Ca^2+^_cyt_ excursions like untreated control cells (Fig. 1, Bi and Bii). For quantifying the probability (P) of radiation-induced Ca^+^ oscillations (P_Ca2+-oscill_) in Fig. 2 A, we consider only repetitive Ca^2+^_cyt_ spikes (five or more spikes) that occurred ≥10 min after start of image acquisition as oscillations. Based on these criteria, the analysis shows that the probability of observing Ca^2+^ oscillations in a population of untreated control cells is only ∼0.1. In contrast, after irradiation with a dose of 5 Gy, the probability of detecting long-lasting Ca^2+^ oscillations increased to P = 0.56.

**Video 1.** **Continuous confocal live-cell imaging of Fluo-4–loaded Jurkat cells.** Cells were exposed to 5 Gy x rays, and image acquisition (one every 5 s) started 5 min after irradiation. The first responsive cell shows blinking 5 min after start of the recording. Over time, ∼65% of the cells in the optical field showed characteristic Ca^2+^_cyt_ oscillations. Time in h:min.

Experiments were repeated over a range of x-ray doses from 0.5 to 5 Gy. These treatments also elicited delayed Ca^2+^_cyt_ oscillations with a high probability (Fig. 2 A). The lag time between x-ray stimulation and onset of Ca^2+^_cyt_ signaling events varied considerably from one cell to another. In 70% of the stimulated cells with 5 Gy, the first detectable Ca^2+^ peak occurred between 10 min (fastest) and 72 min (slowest) after x-ray exposure. A plot of the average lag times as a function of x-ray dose indicates that this value did not significantly change with the stimulation dose (Fig. 1 D). A frequency analysis further reveals that Ca^2+^_cyt_ oscillates in response to x rays with 2–4 mHz (Fig. 1 E). This value remains constant within the large scatter of frequency over the range of irradiation doses.

The fluorescence intensity traces depicted in Fig. 1 C show that the amplitude of the Ca^2+^_cyt_ excursions increases gradually with a saturation kinetic after onset of the oscillations. The peak values of the maximal Ca^2+^_cyt_ excursions are a function of the stimulation doses. From a fit of the data with a single saturating exponential function,

$$Y=F_{\max}\left(1-e^{-x/k}\right)+F_{0},$$ (1)

where *F*_max_ is the maximal increase in peak fluorescence, *F*_0_ the background fluorescence of the control, and *k* the dose for half-maximal increase in fluorescence; the half-maximal Ca^2+^_cyt_ peak is achieved by 1.5 Gy (Fig. 1 F). Notably, the maximal amplitude of Ca^2+^_cyt_ oscillations in Jurkat cells irradiated with 5 Gy x rays was in the same range, but still below the corresponding peak Ca^2+^_cyt_ value measured in the presence of ionomycin (Fig. 1 F). Because the fluorescence intensity of the Fluo-4 dye saturates at ∼1 µM (Dustin, 2000; Gee et al., 2000), we can assume from these data that the x-ray–induced Ca^2+^ oscillations reach peak values close to 1 µM. This value is similar to the amplitude of Ca^2+^_cyt_ oscillations elicited by mitogens in human T cells (Lewis and Cahalan, 1989).

### Ca^2+^_cyt_ oscillations can be suppressed by buffering external Ca^2+^ and by blocking Ca^2+^ influx

Stimulus-induced Ca^2+^_cyt_ oscillations can originate from a release of Ca^2+^ from internal stores or from entry via a plethora of Ca^2+^-permeable channels in the PM of T cells (Trebak and Kinet, 2019). To test the contribution of PM channels to this process, experiments similar to those in Fig. 1 B were repeated in a nominally Ca^2+^-free extracellular solution by the addition of 5 mM EGTA to the buffer. In these experiments, the probability of finding Ca^2+^_cyt_ oscillations was reduced to that of untreated control cells (Fig. 2 A). We further measured the mean fluorescence from single cells over a time window of 60–120 min in control cells and after x-ray exposure with and without indicated treatments; this measurement pools data from cells with Ca^2+^_cyt_ oscillations and cells with a constant Ca^2+^_cyt_ level. The value was significantly higher in cells treated with 5 Gy x rays than that of the sham-irradiated control group (Fig. 2 B). In the presence of EGTA, this value was greatly reduced and only two times higher than the control. The results of these experiments show that the absence of external Ca^2+^ abolishes radiation-induced Ca^2+^_cyt_ oscillations but still allows some steady increase in Ca^2+^_cyt_. The results of these experiments suggest a calcium influx via PM channels as the main trigger of Ca^2+^_cyt_ oscillations in irradiated cells. The remaining steady increase of Ca^2+^_cyt_ furthermore suggests that x-irradiation may have a negative impact on the mechanisms of Ca^2+^_cyt_ buffering and Ca^2+^_cyt_ clearance.

To further test the relationship between Ca^2+^ influx and Ca^2+^ oscillations, experiments were repeated in a buffer containing Ca^2+^ ± 5 µM gadolinium (Gd^3+^), a broad inhibitor of Ca^2+^-permeable channels in T cells (Biagi and Enyeart, 1990; Adding et al., 2001). In the presence of the inhibitor, the probability of radiation-induced Ca^2+^_cyt_ oscillations was reduced close to that in control cells (Fig. 2 A); as in experiments with EGTA, in this case a constant increase in Ca^2+^_cyt_ was still visible (Fig. 2 B). Together, these data underscore the impact of radiation on Ca^2+^_cyt_ and the importance of Ca^2+^ influx for the radiation-induced signaling cascade, leading to characteristic Ca^2+^_cyt_ oscillations. The data further suggest that x-ray irradiation stimulates some additional mechanisms, which cause a steady increase in Ca^2+^_cyt_. The finding that the latter was abolished by neither depletion of external Ca^2+^ nor blocking Ca^2+^ channels with Gd^3+^ suggests that at least some of this Ca^2+^ originates from internal stores.

### Ca^2+^ oscillations are mediated by STIM1/Orai1 activation

**Figure 3.**
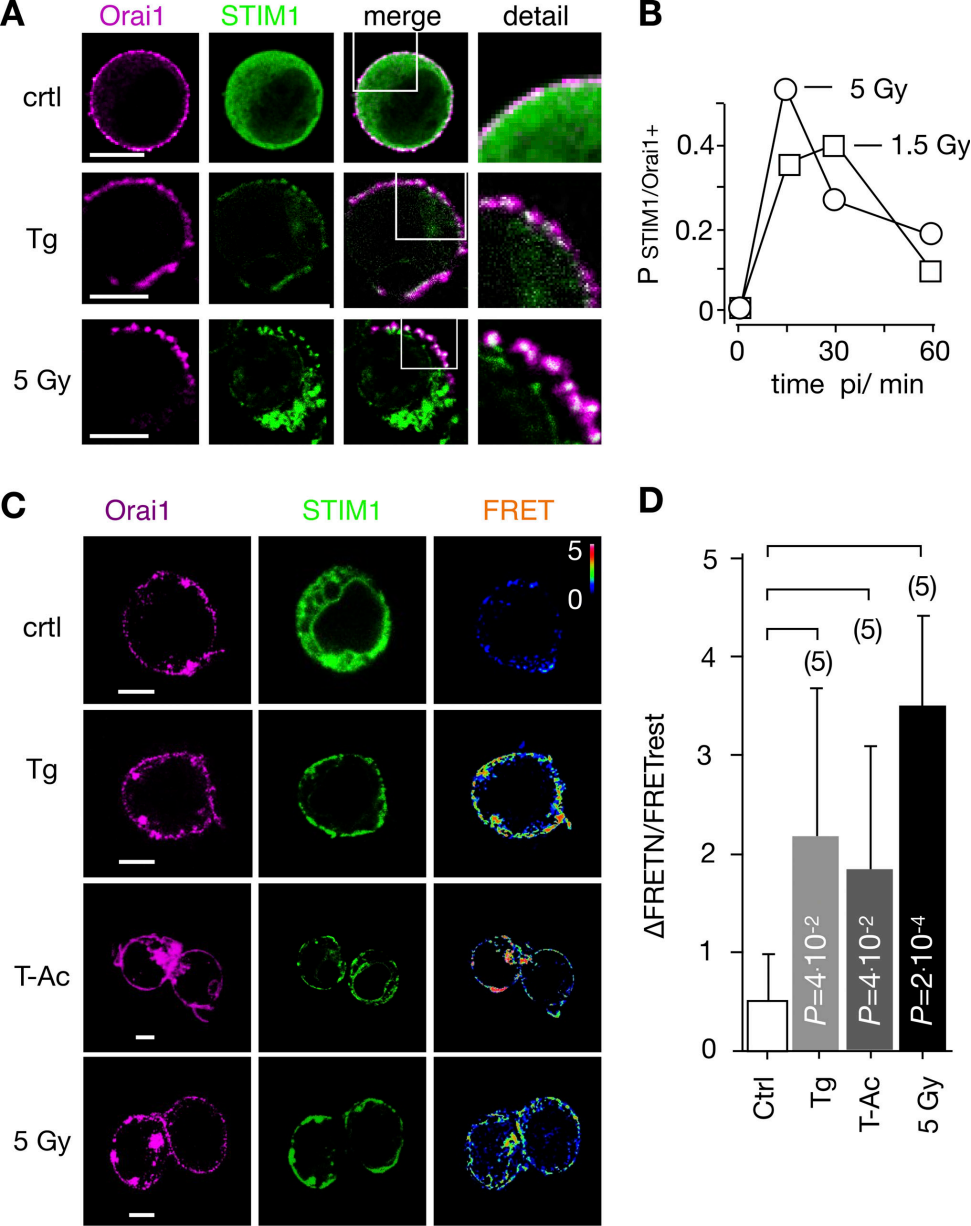
**IR triggers Ca**^**2+**^ **regulated STIM1/Orai1 CRAC channel formation. (A)** Distribution of endogenous Orai1 (magenta, first column) and STIM1 (green, second column) in Jurkat cells immune-stained with Alx647 and Alx488, respectively. Overlays of green and magenta images with magnification of indicated areas are shown in third and fourth columns. Fixed cells were obtained from untreated/nonirradiated control cells (top row), cells treated for 15 min with 2 µM Tg (central row), or cells 15 min after 5-Gy x-ray exposure (bottom row). **(B)** Probability of finding, in a population of Jurkat cells, positive clustering of STIM1/Orai1 (P_STIM1/Orai1+_) after irradiation with 1.5 Gy (squares) or 5 Gy (circles). Criteria for cluster detection are specified in Materials and methods. For each condition, ≥282 cells were analyzed. **(C)** Representative confocal images of same cells with fluorescent donor molecule Orai1::eCFP (magenta, first column), acceptor molecule STIM1::eYFP (green, second column), and heatmaps of the resulting FRET signals (third column) 15 min after treatment. Images are from untreated cells (control), cells incubated with 2 µM Tg, 25 μl/ml ImmunoCult Human CD3/CD28/CD2 T-Ac, or irradiated with 5 Gy. All three treatments generate a visible FRET-signal in the PM. Scale bars, 10 μm. **(D)** Mean FRET signal (±SD, *n* ≥ 5) from PM of cells as in C: untreated/nonirradiated control cells (crtl), cells 5 min in 2 μM Tg, 15 min in 25 μl/ml T-Ac, or 20 min after irradiation with 5 Gy. Statistical differences between treatments were analyzed by unpaired Student’s *t* test, and respective P values are given in the figure.

The major mechanism for Ca^2+^ entry to T cells’ interior is provided by the SOCE pathway (Trebak and Kinet, 2019). In this system, the calcium level of the ER is monitored by the ER Ca^2+^ sensor STIM1. Upon store depletion, STIM1s aggregate and move to contact points with the PM, where they interact and activate the Orai1 channel subunit (Zheng et al., 2018). The active Orai1/STIM1 complex, which assembles to the CRAC channel (Gudlur and Hogan, 2017), is necessary and sufficient to support SOCE. To test the involvement of this pathway in radiation-induced T cell stimulation, we monitored the dynamic distribution of Orai1 and STIM1 in Jurkat cells and PBMCs. Cells were therefore fixed for immunostaining 15, 30, and 60 min after x-ray exposure or 15 min after their activation with 2 μM Tg. The representative images in Fig. 3 A and Fig. S1 show the typical distribution of the two CRAC channel components in unstimulated cells. The Orai1 channel subunit is evenly distributed in the PM, while the STIM1 proteins generate a diffuse signal throughout the cytoplasm (Fig. 3 A, top row). After activating cells with Tg, STIM1 proteins are translocated from the cytosol to the PM, where they colocalize with the Orai1 proteins in distinct clusters (Fig. 3 A, middle row).

The same redistribution of STIM1 and Orai1 emerged after exposing Jurkat cells to doses of 1.5 and 5 Gy. The STIM1 signal disappears from the cytosol and concentrates in the PM, where it forms clusters with Orai1 (Fig. 3 A, bottom row). Already 15 min after irradiation, a maximum clustering of both proteins was observed in ∼50% of the cells analyzed before clustering gradually decreased (Fig. 3 B). A similar transient clustering of STIM1 and Orai1 was evident with 1.5-Gy x-ray exposure.

To confirm x-ray–triggered aggregation of STIM1 and Orai1, we coexpressed STIM1::eYFP and Orai1::eCFP in Jurkat cells and measured protein–protein interactions by FRET. Data in Fig. 3, C and D, show that the FRET signal is small in untreated control cells. It significantly increased in cells stimulated with 2 μM Tg or 25 μl/ml ImmunoCult human CD3/CD28/CD2 T-Ac (short T-Ac). An even larger energy transfer was measured in cells exposed to a dose of 5 Gy x rays. Together, these data confirm that STIM1 and Orai1 interact in response to x-ray exposure.

**Figure 4.**
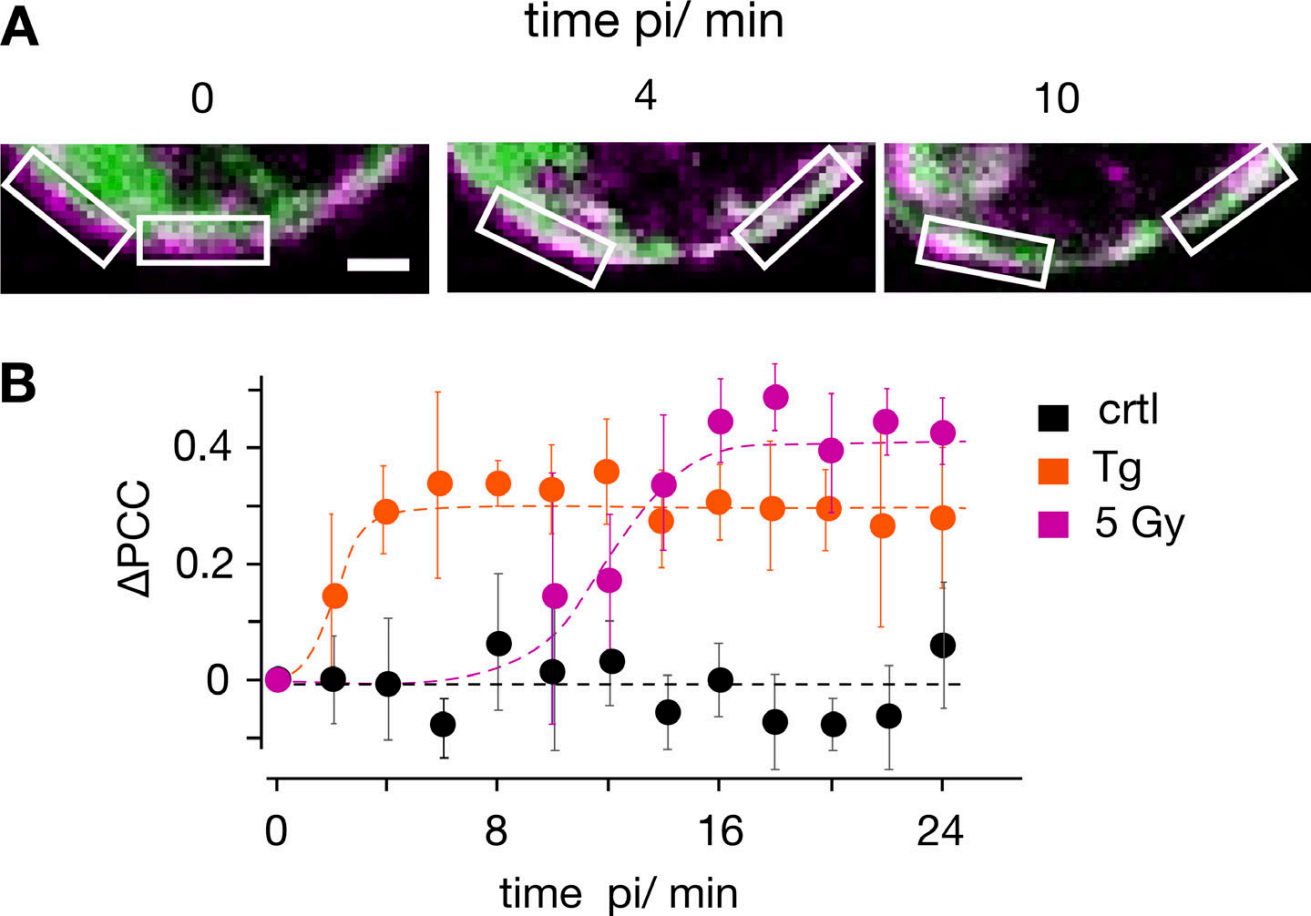
**Time course of stimulus-induced STIM1/Orai1 colocalization. (A)** Fluorescent images of a representative Jurkat cell cotransfected with STIM1::eYFP (green) and Orai1::eCFP (magenta) with focus on PM/cytosol interface. Images were taken before (0 min) and 4 and 10 min after treating cells with 10 μM Tg. White boxes show ROIs in membrane/cytosol interface for calculating PCC value of the two fluorescent markers. **(B)** Change in PCC (∆PCC) for colocalization of STIM1::eYFP and Orai1::eCFP. Data obtained from confocal live-cell real-time acquisition of Jurkat cells as in A, heterologously expressing the two proteins. In five independent experiments a mean PCC of 3.8 ± 0.08 was estimated from 18 untreated control cells (triangle). Changes in PCC values over time from untreated and treated cells (circles) are shown as deviation from this control value (∆PCC). The PCC values of untreated cells remain at the same level (ctrl, black) but increase with different kinetics in cells stimulated with 2 µM Tg (orange) or 5 Gy x rays (5 Gy, magenta). The data were fitted with logistic equation (Eq. 2, solid lines) yielding the following times for half-maximal increase in STIM1/Orai1 colocalization: 2 min for Tg and 12 min for x ray. Data for the three conditions are mean values ± SD from *N* ≥ 4 independent experiments with *n* ≥ 4 cells each. Scale bars, 2.5 μm.

To estimate the time course for stimulus-induced STIM1/Orai1 clustering, we coexpressed both fluorescent tagged proteins in Jurkat cells and monitored by live-cell imaging the dynamics of colocalization between STIM1 and Orai1. The images in Fig. 4 A show a close-up of the PM with a merger of STIM1::eYFP (green) and Orai1::eCFP (magenta). The series of images exhibit a distinct separation of the two signals before stimulation with 10 μM Tg. After stimulation, the green signal (STIM1) moved from the cytosol to the PM, which hosts Orai1::eCFP, shown in magenta. To quantify the stimulus-induced redistribution of STIM1-associated fluorescence to the PM, we measured the Pearson correlation coefficient (PCC) for the two fluorescent signals in defined regions of interest (ROIs) over the PM (Fig. 4 A). The quantitative analysis shows that mean colocalization values in untreated control cells were low (PCC = 0.38 ± 0.08 in 18 cells). This value was used as a reference for estimating a progressive colocalization between STIM1 and Orai1 over time. The data in Fig. 4 B show that the PCC value of untreated control cells did not change over a period of 24 min. When cells were stimulated with either Tg or 5 Gy x rays, the PCC value increased over the time window rapidly (Tg) or slowly (x ray) to new plateau values. To estimate the time course of changes in PCC values the mean data were fitted by a logistic equation:

$$Y=a/\left[1+e^{-k\times \left(x-\mathrm{xo}\right)}\right],$$ (2)

where *a* is the maximal PCC value, *k* the rate of increase, and *x*_*o*_ the time of maximal increase. The half-maximal increase in colocalization was achieved in 2 and ∼12 min after stimulation with Tg or 5 Gy x rays, respectively (Fig. 4 B).

**Figure 5.**
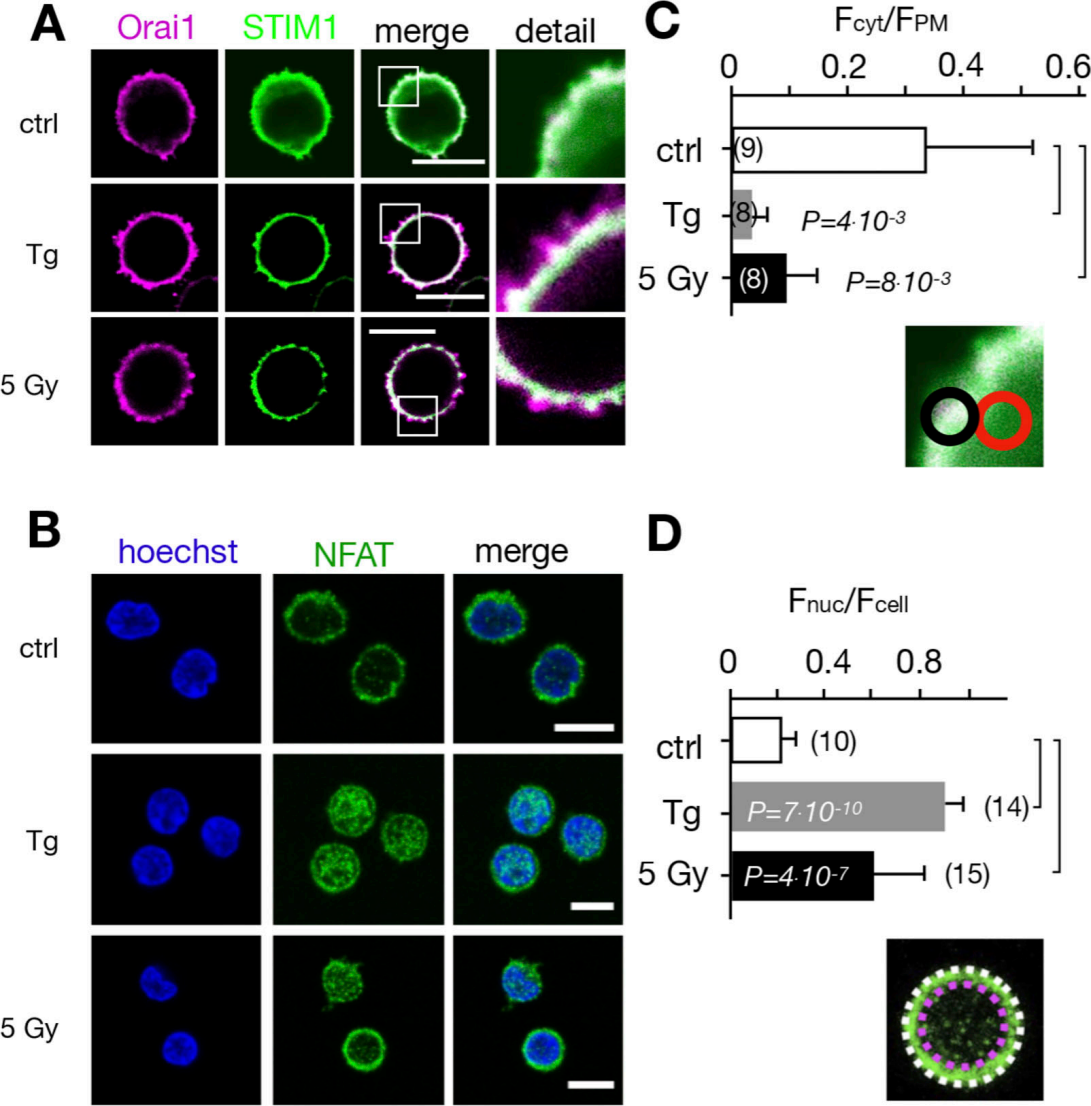
**Calcium-dependent SOCE/NFAT pathway is activated by IR in naive T-lymphocytes. (A)** Distribution of endogenous Orai1 (magenta, first column) and STIM1 (green, second column) in fixed PBMCs immunostained with secondary antibodies Alx488 and Alx647, respectively. A merge of the two channels is shown in the third column, with higher magnification of marked areas in the fourth column. The merge of untreated control cells additionally shows the nucleus stained with Hoechst DNA dye (blue). Images show cells that were fixed as untreated/nonirradiated control cells (ctrl, top row) and cells fixed 15 min after treatment with 2 µM Tg (second row) or after exposing cells to 5 Gy (third row). **(B)** Mean ratio (± SD, number of cells in brackets) of green fluorescence in ROI (inset image, red circle) in cytosol divided by fluorescence in ROI in direct vicinity over PM (black circle). Data from untreated control cells (ctrl) as well as cells exposed to 2 μM Tg or 5 Gy x rays 60 min after treatment. **(C)** Confocal images of PBMCs showing nucleus stained with Hoechst DNA dye (blue, first column) and endogenous NFATc2 (green, second column) stained with Alx488. Overlay of both columns is shown in third column. Cells were fixed immediately (untreated/nonirradiated control, top row), 15 min after 2 µM Tg Ca^2+^ store depletion (second row) or 60 min after x-ray exposure with 5 Gy (third/fourth row). All scale bars, 10 μm. **(D)** Mean ratio (± SD, number of cells in brackets) of GFP fluorescence in nucleus (inset image, magenta circle) divided by fluorescence of total cell (white circle). Statistical differences between treatments in B and D were analyzed by unpaired Student’s *t* test, and respective P values are given in the figure. Source data are available for this figure: SourceData F5.

To test whether STIM1/Orai1-mediated *SOCE* is crucial for the radiation-induced Ca^2+^ oscillations, we repeated experiments as in Fig. 1 B with established CRAC channel inhibitors. Cells pretreated with either 10 µM Synta66 (*N* = 6) or 5 µM Pyr6 (*N* = 6) exhibited in the presence of both blockers a strongly reduced propensity of Ca^2+^ oscillations after x-ray exposure (Fig. 2 A). To further test the crucial role of CRAC channels in this signaling cascade, we generated a knockout of Orai1 in Jurkat cells by CRISPR-Cas9. The resulting mutant cells exhibit no-longer-detectable levels of Orai1 protein in Western blots (Fig. S2 B) or immunofluorescence staining (Fig. S1 E) and accordingly show a drastically reduced SOCE profile after Tg stimulation (Fig. S2 A). Fig. S2 A shows that the peak and the plateau values of Tg-induced Ca^2+^ influx are greatly reduced in Orai1 knockout (KO) cells compared with WT cells. Hence, the knockout of the Orai1 isoform is sufficient to greatly reduce SOCE in mutant cells. When knockout cells were exposed as in Fig. 1 B to 5 Gy x rays, we no longer observed Ca^2+^ oscillations (Fig. 2 A), while these cells still exhibited the same elevated increase in Ca^2+^_cyt_ level that was observed in the presence of the CRAC channel blockers (Fig. 2 B). The results of these experiments underpin that radiation-stimulated Ca^2+^_cyt_ oscillations are indeed initiated by clustering of STIM1 and Orai1 and by a consequent SOCE.

### The STIM/Orai activation pathway is also activated in naive T-lymphocytes

Jurkat cells are a leukemic T cell line, which serves as a model system for uncovering the basic signaling events engaged in T cell activation (Abraham and Weiss, 2004). To test whether the irradiation-triggered Ca^2+^ signaling cascade also occurs in naive T cells, we repeated the experiments in Fig. 3 with PBMCs. The images in Fig. 5 A indicate that the cytosol volume of nonstimulated lymphocytes is even smaller than that of Jurkat cells, which makes it more difficult to detect a translocation of STIM1 from the cytosol to the membrane-resident Orai1. A comparative analysis nonetheless shows that the Orai1 and STIM1 distribution remains uniform in unstimulated control cells, whereas they exhibit a distinct clustering after stimulation with 2 μM Tg. The same clustering is also evident after exposing cells to 5 Gy x rays (Fig. 5 A). The visual impression from the representative images was confirmed by quantitative analysis (Fig. 5 B), which shows that x-ray irradiation and Tg treatment favor the removal of the green fluorescence from the cytosol. The results from these experiments underscore that the IR-induced Ca^2+^ signaling cascade is a general response of resting T cells.

### Radiation-induced STIM1/Orai1 activation is preceded by depletion of Ca^2+^ stores

**Figure 6.**
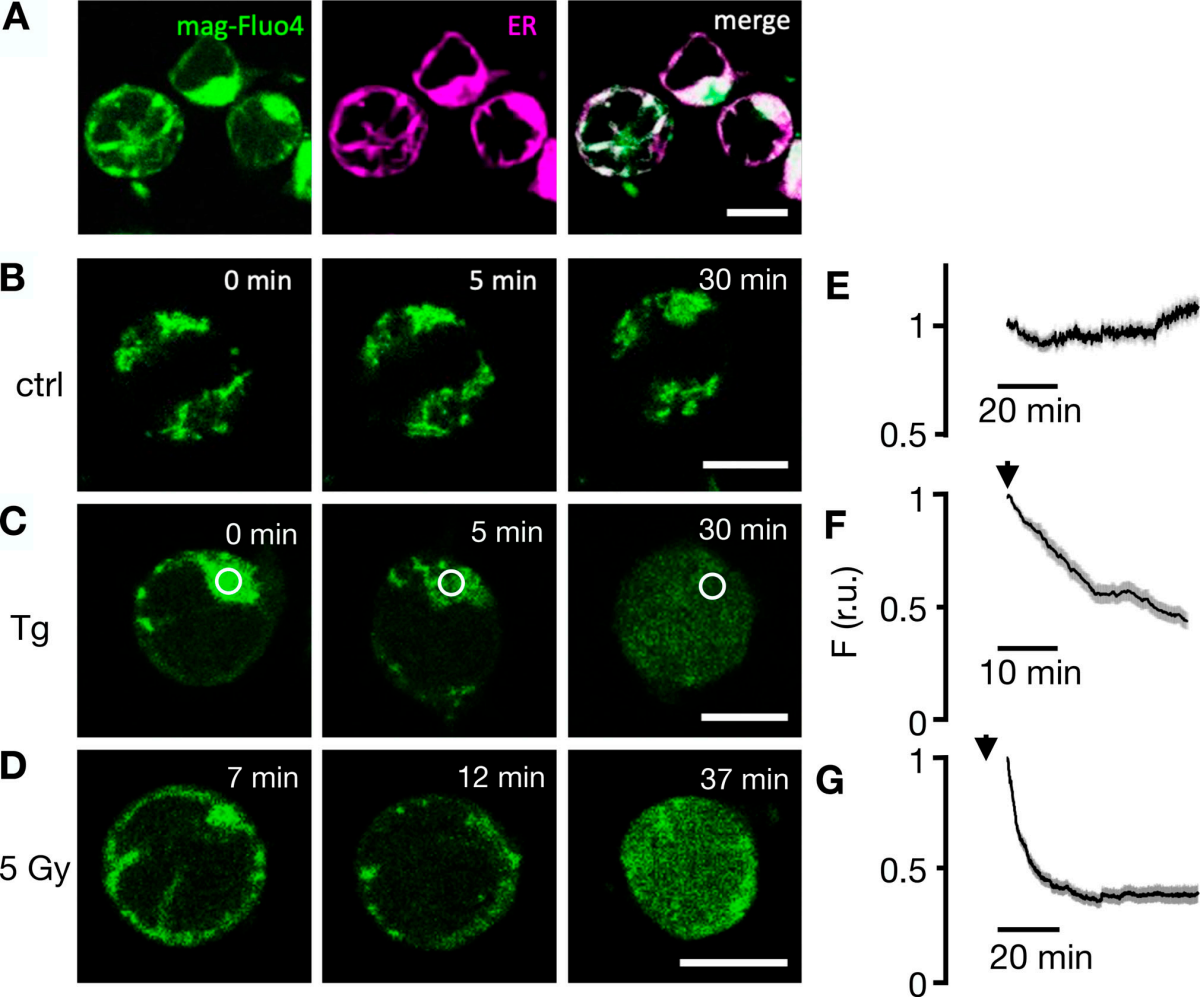
**Fluorescent sensor Mag-Fluo-4 reports depletion of ER Ca**^**2+**^ **in Jurkat cells as a response to irradiation. (A)** Fluorescent images of representative Jurkat cells loaded with Mag-Fluo-4 (green, first column) and stained with ER tracker red (magenta, second column) exhibit colocalization of both fluorescent signals in merged image (third column). **(B)** In untreated control cells, the Mag-Fluo-4 fluorescence in the ER remains constant. **(C)** Challenging cells with 2 µM Tg (D) or irradiating cells with 5 Gy x rays elicits progressive decrease in Mag-Fluo-4 fluorescence in ER with concomitant increase in cytosol. Times in images denote time point of imaging after respective treatment. Corresponding mean values of relative Mag-Fluo-4 fluorescence (± SD) in ROI (white circle in C) over ER in untreated control cells (E), cells exposed to 2 μM Tg (F), and cells irradiated with 5 Gy x rays (G). Start of imaging after treatments are indicated by an arrow. Data in E–G were normalized to fluorescence values at start of analysis from ≥35 cells per treatment in ≥7 experiments. Scale bars, 10 µm.

Next, we addressed the question whether the observed STIM1/Orai1 clustering is a consequence of an x-ray–triggered depletion of intracellular Ca^2+^ stores. Jurkat cells were therefore loaded with the fluorescent dye Mag-Fluo-4. Because of a combination of distinct affinities for Ca^2+^ and Mg^2+^ and due to the prevailing concentrations of both divalent cations in the ER, the fluorescent dye can be used for monitoring changes in the high Ca^2+^ concentration in the ER, but not reliably for changes in the cytosol (Rossi and Taylor, 2020; Lebeau et al, 2021). Fig. 6 A shows representative images of Jurkat cells loaded with Mag-Fluo-4 and stained with ER tracker red. The overlay of both images exhibits a bright Mag-Fluo-4 signal in association with the ER, confirming that the sensor enters this organelle with its high Ca^2+^ concentration. While the fluorescent signal remains high and constant in the ER of control cells (Fig. 6, B and C), it decreases in the ER and concomitantly increases in the cytosol (Fig. 6, D and E) after addition of 2 μM Tg to the bath medium. The measured decrease in Mag-Fluo-4 fluorescence is consistent with the expected Tg-induced depletion of Ca^2+^ from these stores (Lebeau et al., 2021). After confirming that Mag-Fluo-4 can be used for monitoring Ca^2+^ store depletion in Jurkat cells, we followed its fluorescence in cells after irradiation with 5 Gy x rays. In nine experiments, we observed that the Mag-Fluo-4 signal remained in 40% of the cells after irradiation on the level of untreated control cells. In the remaining 60% of cells, the Mag-Fluo-4 fluorescence decreased progressively after irradiation with 5 Gy. The representative images and the corresponding quantitative analysis (Fig. 6, F and G) of the responsive cells show that irradiation with x ray also elicits a gradual decrease in Mag-Fluo-4 fluorescence in the ER of exposed cells; this effect of Ca^2+^ store depletion was already visible at the start of imaging 7 min after irradiation. The images show that the Tg- and x-ray–induced changes in Mag-Fluo-4 fluorescence are a combination of Ca^2+^ store depletion and concomitant increase in Ca^2+^_cyt_. Because of the mixed nature of this signal, the recorded decay of fluorescence intensity cannot be taken as a direct measure of store depletion kinetics. Still, the data indicate that the x-ray–induced depletion of Ca^2+^ stores and the formation of STIM1/Orai1 clusters (Fig. 5) occur during the same time window of ∼20 min after stimulation. Collectively, the results of these experiments suggest that x-ray irradiation evokes in a yet-unknown manner a depletion of Ca^2+^ stores in Jurkat cells, which may in turn promote the observed clustering of STIM1/Orai1 and CRAC channel activation.

### Ca^2+^ signaling cascade results in a translocation of NFAT to the nucleus

Ca^2+^ entry via the SOCE pathway is the main source for activation of the transcription factor isoforms of NFAT (Dolmetsch et al., 1998; Tomida et al., 2003). The stimulus-induced and Ca^2+^-dependent nuclear translocation of NFAT is instrumental for subsequent cytokine expression, proliferation, and immune competence (Crabtree and Olson, 2002). Notably, the frequency of the x-ray–induced Ca^2+^_cyt_ oscillations of ∼4 mHz in Jurkat cells (Fig. 1 E) is typical for a signaling cascade, which elicits the activation of the NFAT pathway (Smedler and Uhlen, 2014).

**Figure 7.**
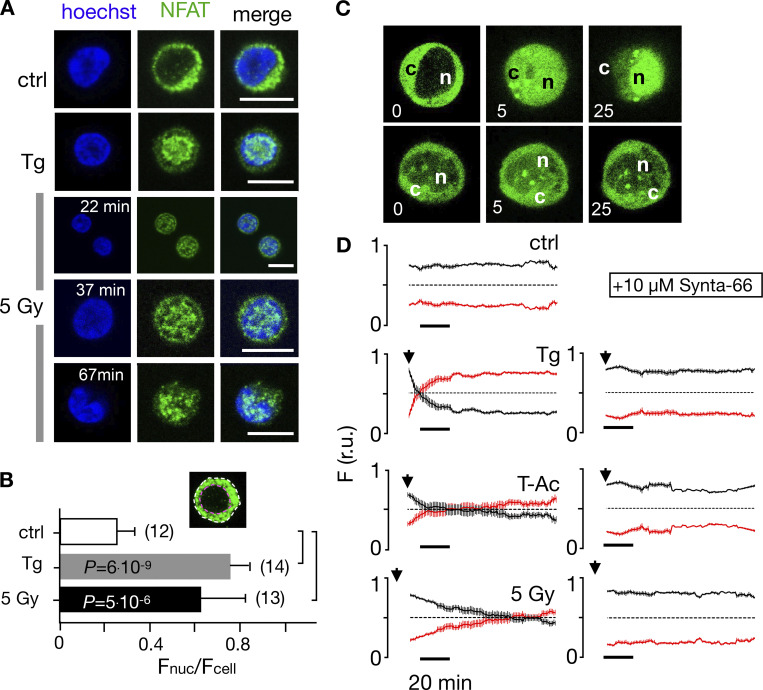
**Nuclear translocation of Ca**^**2+**^**-dependent NFAT in Jurkat cells. (A)** Confocal images of Jurkat cells in which nucleus was stained with Hoechst DNA dye (blue, first column) and endogenous NFATc2 immunostained with secondary antibody Alx488 (green, second column). The third column shows a merge of blue and green channels. Cells were fixed immediately in untreated/nonirradiated control cells (ctrl, first row), 15 min after 2 µM Tg Ca^2+^ store depletion (Tg, second row), or 15, 30, and 60 min after x-ray exposure with 5 Gy (three bottom rows). **(B)** Mean ratio (± SD, number of cells in brackets) of GFP fluorescence in nucleus (inset, magenta circle) divided by fluorescence of total cell (white circle). Statistical differences between treatments were analyzed by unpaired Student’s *t* test, and respective P values are given in the figure. **(C)** Live-cell imaging of nuclear import of transiently expressed NFATc2-GFP from cytosol (c) to nucleus (n) in Jurkat cells after stimulation with 2 μM Tg in absence (top) or presence (bottom) of 10 µM CRAC channel inhibitor Synta66. Numbers indicate time in minutes after treatment. **(D)** Kinetic analysis of NFATc2-GFP nuclear translocation from cytosol (black) to nucleus (red). Data are from confocal imaging of Jurkat cells in untreated control condition (crtl), with 2 µM Tg in 25 μl/ml activator (T-Ac), or after 5 Gy x-ray exposure (5 Gy). Data were obtained without (left) and with (right) 10 µM Synta66. Each time course diagram is the mean ± SE of ≥12 individually measured cells. Addition of Tg and T-Ac as well as time of x-ray irradiation are indicated by arrows in D. Relative fluorescence values for NFAT in cytosol (black) and nucleus are (red) were normalized to 1. Source data are available for this figure: SourceData F7.

To test if the NFATc2 pathway, which is the most prominent in T cells, is indeed activated by IR, we monitored nuclear translocation of endogenous NFATc2 labeled with Alx488. The representative images and the corresponding quantitative analysis depicted in Figs. 5 and 7 show that the transcription factor is primarily located in the cytosol in naive T cells (Fig. 5, C and D) and unstimulated Jurkat cells (Fig. 7, A and B). Stimulation with 2 μM Tg favors a translocation of NFAT into the nucleus in a major population of cells tested (Fig. 2 C). A similar nuclear translocation is induced in 72% of Jurkat cells irradiated with 5 Gy (Figs. 7 and 2 C).

To analyze stimulus-induced translocation of NFAT, we monitored Jurkat cells heterologously expressing GFP-tagged NFAT by live-cell imaging in real time (Fig. 7 C and Video 2). Again, analyses of NFAT accumulation in the nucleus highlights distinct response times to different stimuli: after Tg stimulation, half of the NFAT translocation (in 97% of all cells monitored) is achieved after 8 ± 1.5 min. With T-Ac, it takes 21 ± 6.5 min (in 89% of the cells), and with 5 Gy irradiation, 77 ± 5 min (in 67% of all cells) for the same response (Fig. 7 D). The nuclear translocation of NFAT is causally related to the activation of CRAC channels for all three stimuli, as treating cells with 10 µM of the CRAC channel inhibitor Synta66 abolishes its nuclear translocation (Fig. 7, C and D; and Fig. 2 C) in all cases.

**Video 2.** **Confocal live-cell imaging of Jurkat cells transiently expressing GFP-tagged NFAT.** In the resting state, GFP fluorescence is predominantly visible in the cytosol. 8 min after stimulation with 5 Gy x rays, NFAT-GFP fluorescence alters its location and progressively moves over a period of ∼30 min completely into the nucleus. Time in h:min; images taken with a frame rate of one/5 s.

**Figure 8.**
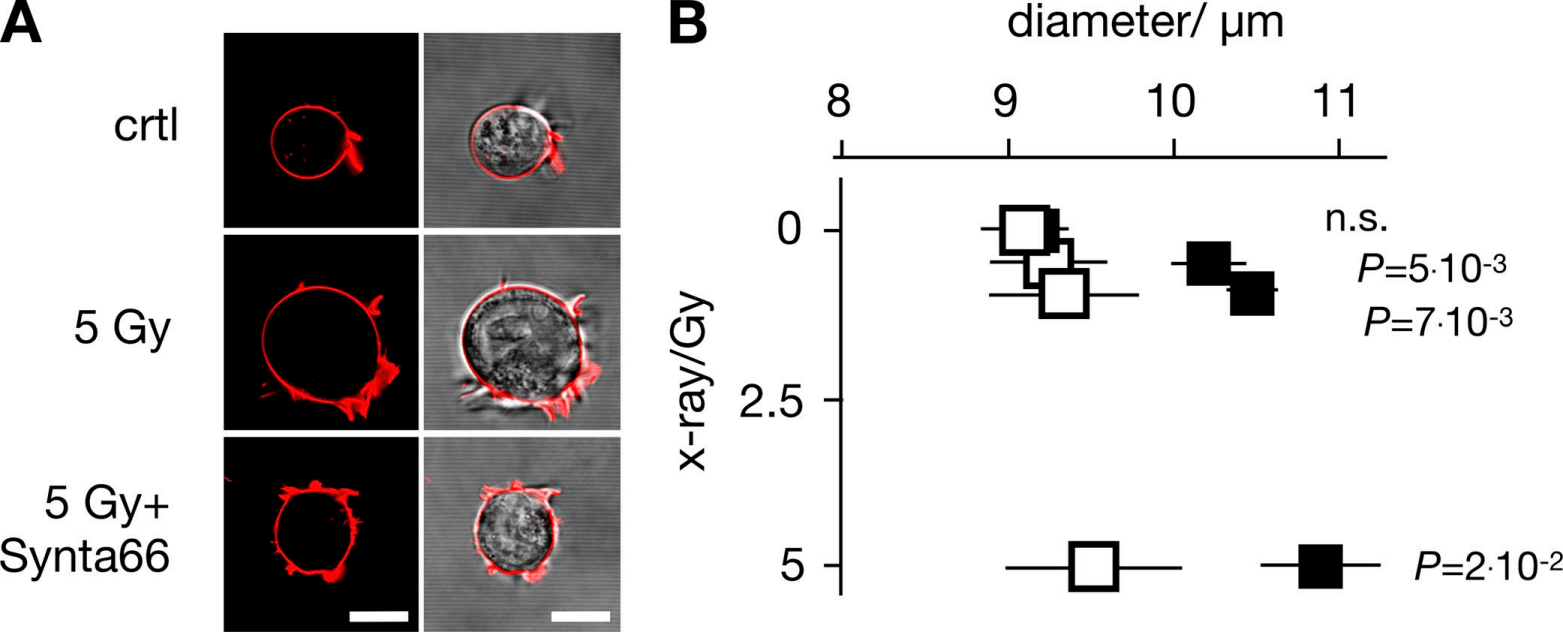
**CRAC channel blocker Synta66 inhibits x-ray–stimulated increase in Jurkat cell diameter. (A)** Jurkat cells exhibit a dose-dependent increase in diameter 24 h after irradiation, which can be abolished by 10 µM CRAC channel inhibitor Synta66. Representative images (left, confocal image of fluorescent stained PM [red]; right, overlay of fluorescent and bright field image) of individual Jurkat cells. Images were taken 24 h after start of experiment with an untreated control cell (ctrl), a cell exposed to 5 Gy x rays alone (5 Gy) or with 10 µM Synta66 (5 Gy + Synta66). **(B)** Mean diameters ± SD of >300 cells per treatment (*N* ≥ 5 independent experiments) 24 h after exposure to 0–5 Gy x rays in the absence (black square) or presence (open square) of 10 µM Synta66. Statistical differences between treatments ± Synta66 were analyzed by unpaired Student’s *t* test, and respective P values are given in the figure. All scale bars, 10 μm.

Finally, to examine the consequence of CRAC channel activation on the physiological response of Jurkat cells, we monitored the effect of 10 µM Synta66 on irradiation-induced increase in cell diameter, since the morphological change is part of the radiation-induced immune response (Voos et al., 2018). The data depicted in Fig. 8 indicate that the >1,000 analyzed cells have a uniform diameter (9.4 ± 0.2 µm) under control conditions. Over 24 h, this diameter does not change in the presence of 10 µM Synta66 (9.2 ± 0.1 µm) or in the absence of Synta66 (9.3 ± 0.3 µm). X-ray irradiation with three different doses (0.5, 1.5, and 5 Gy) caused a dose-dependent, significant increase in cell diameter of ∼4–21%. This increase in cell size was largely abolished in the presence of the CRAC channel blocker (Fig. 8). The results of these experiments suggest that the morphological change in Jurkat cells in response to x-ray stimulation is an endpoint of a signal transduction cascade, which involves SOCE via STIM1/Orai1 clustering.

## Discussion

Stimulus-induced Ca^2+^ signaling cascades are key events in the activation of T cells. Triggered by antigen binding to the T cell receptor (Bryceson et al., 2006), a downstream signaling cascade is initiated that promotes the release of Ca^2+^ from internal stores and eventually the activation of SOCE. In this process, Ca^2+^ enters the cytoplasm primarily via CRAC channels, generating Ca^2+^_cyt_ oscillation patterns with distinct durations and amplitudes according to the stimulus (Salazar et al., 2008; Parekh, 2011). These dynamic changes in the concentration of the second messenger molecule are finally decoded by cytosolic Ca^2+^-dependent target enzymes, including kinases, phosphatases, and transcription factors such as NFAT (Cifuentes et al., 1993; Kohout et al., 1994; Stemmer and Klee, 1994). With this network of signaling steps, T cells achieve precise control over essential lymphocyte functions such as cytokine production, proliferation, differentiation, and antigen-dependent cytotoxicity. In the present study, we indicate that the signaling cascades involving CRAC channel activation, SOCE-mediated Ca^2+^_cyt_ excursions, and translocation of NFAT from the cytosol to the nucleus can be triggered in a population of about half of the T cells examined by clinically relevant doses of ionizing irradiation. In this regard, IR elicits effects comparable to Tg and a well-established T-Ac (ImmunoCult Human CD3/CD28/CD2 short T-Ac). Further, the finding that specific inhibitors of CRAC channels or the downregulation of Orai1 by CRISPR-Cas9 inhibit or even abolish the crucial Ca^2+^_cyt_ oscillations underpins that they all use SOCE as the major Ca^2+^ entry pathway. This is additionally supported by the finding that the CRAC channel blocker Synta66 abolishes subsequent downstream effects like the translocation of NFAT and the increase in cell diameter. Other Ca^2+^ channels in T cells seem to play no primary role in the IR-induced Ca^2+^ signaling, which regulates these cellular reactions.

It is well established that the form of Ca^2+^_cyt_ signals in T cells determines their cellular response (Parekh, 2011; Bootman and Berridge, 1995; Cahalan, 2009; Song et al., 2012). Depending on the nature and the concentration of the stimuli, the Ca^2+^ signal can exhibit either a sustained increase in Ca^2+^_cyt_ or periods of Ca^2+^_cyt_ oscillations with different frequencies and amplitudes. This frequency- and amplitude-encoded signature of the Ca^2+^_cyt_ oscillations bears information on the subsequent differentiation of T cells; it can induce either cell proliferation or death (Orrenius et al., 2003). Different amplitudes and frequencies also activate different transcription programs in populations of T cells (Dolmetsch et al., 1997). Scrutiny of IR-induced Ca^2+^ oscillations shows that they oscillate independently of the IR doses, with frequencies of 2–4 mHz. This is exactly in the frequency range of antigen-triggered oscillations in T cells that eventually result in activation of NFAT (Dolmetsch et al., 1998; Smedler and Uhlen, 2014). Our finding that IR activates the NFAT pathway in T cells is in good agreement with the view that both stimuli trigger the same signaling pathways.

An interesting finding in the present study is that IR-induced depletion of Ca^2+^ stores and the consequent activation of CRAC channels are not an immediate consequence of radiation exposure; they occur with a delay of several minutes. This delay is well beyond the lifetime of oxygen radicals, including long-lived H_2_O_2_ (Sies, 2017; Mikkelsen and Wardman, 2003), meaning that Ca^2+^ oscillations are not initiated by radiolysis of water or direct peroxidation/oxidation of lipids/proteins. This lack of an immediate impact of IR on Ca^2+^_cyt_ is consistent with previous high-resolution imaging experiments that showed that individual high-energy ions had no immediate impact on the Ca^2+^ concentration along the track of the ions (Du et al., 2008). The current data are not sufficient to explain the primary action of IR and the series of events during the gap between IR exposure and Ca^2+^ store depletion. In this context, however, it is interesting to note that x-ray irradiation was causing—even in the presence of EGTA in the medium—a steady increase in Ca^2+^_cyt_ (Fig. 2 B). Such a negative impact of IR on Ca^2+^_cyt_ buffering and Ca^2+^_cyt_ clearance could eventually contribute to the mechanism of Ca^2+^ store depletion, if it includes a downregulation of the SERCA, e.g., the pump that refills the store with Ca^2+^.

Since immune cells like T cells, which are cycling in the blood, are inevitably exposed to IR during tumor therapy, it is important to understand their response to it. The present study underpins a stimulating role of IR on STIM1/Orai1 cluster formation and a subsequent activation of these CRAC channels for SOCE, not only in a T cell cancer cell line (Jurkat), but also in naive peripheral blood lymphocytes. T-lymphocyte activation is at the forefront of antitumor cytotoxic effects and the regulation of an adaptive immune response in healthy and cancerous tissue (Demaria et al., 2015; Schaue and McBride, 2012). Our findings thus have implications for the understanding of both radiation-associated toxicity in normal tissue and the efficacy of radiation therapy, especially if combined with checkpoint PD-1 and PD-L1 inhibitors in current clinical practice. Further, STIM1 and Orai1 proteins are not restricted to T cells but are also expressed in B cells, as well as in phagocytes such as neutrophilic granulocytes, macrophages, and dendritic cells (Demaurex and Nunes, 2016), where they regulate a multitude of cellular reactions (Vaeth and Feske, 2018). With a more general functional importance of these channel-forming proteins in different types of immune cells, we anticipate that IR activation of CRAC channels will have even a more global importance in the modulation of the immune responses following radiation exposure in tumor therapy.

## Supplementary Material

SourceData F5 is the source file for Fig. 5.

SourceData F7 is the source file for Fig. 7.

SourceData FS1 is the source file for Fig. S1.
