# Supplementary figures and images for: X-ray irradiation triggers immune response in human T-lymphocytes via store-operated Ca2+ entry and NFAT activation
Source: J Gen Physiol. 2022 Apr 13;154(5):e202112865. doi: 10.1085/jgp.202112865 (PMC9011325; doi:10.1085/jgp.202112865)

5A

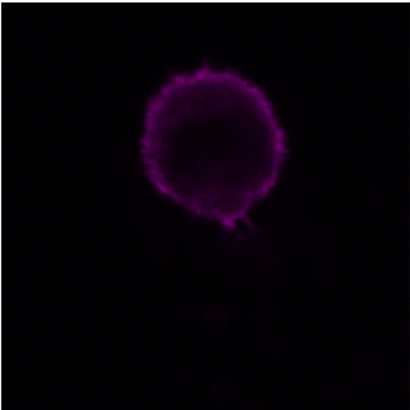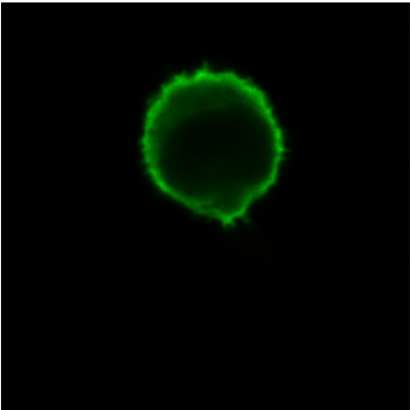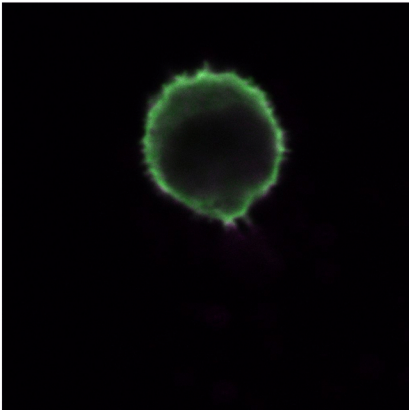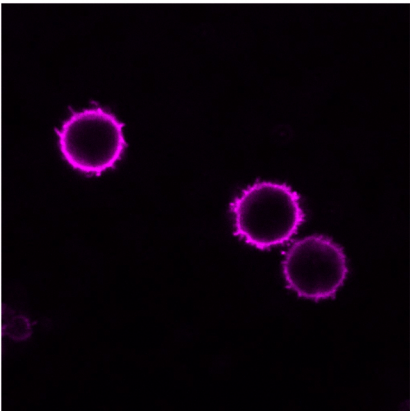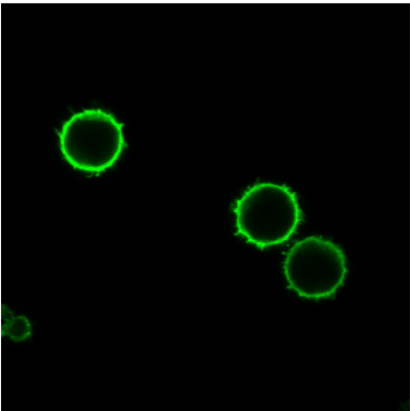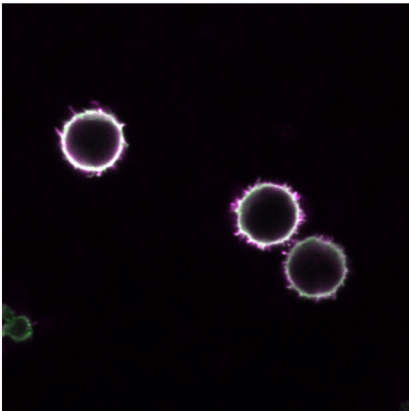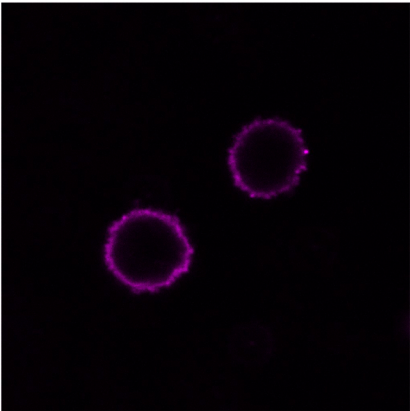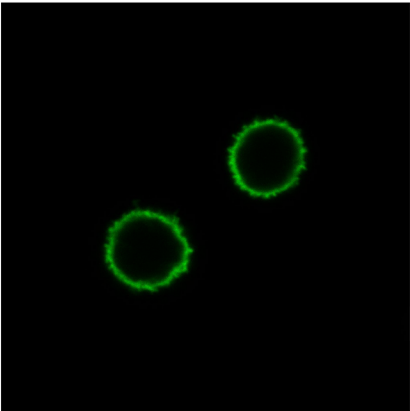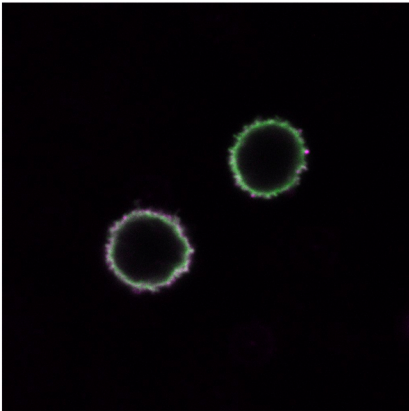

Supplement: SourceData F5 — is the source file for Fig. 5. [file JGP_202112865_SourceDataF5.pdf]

7A

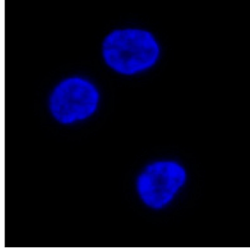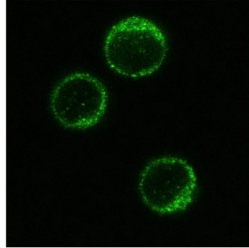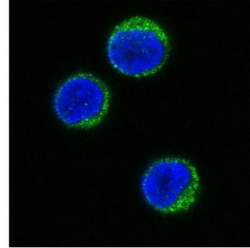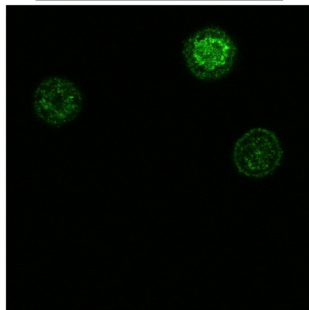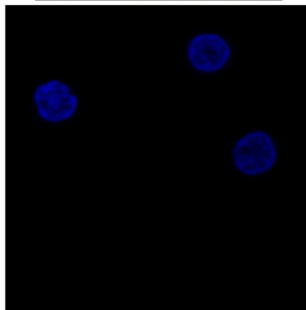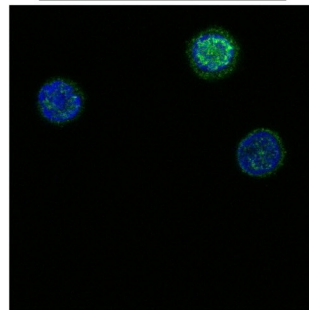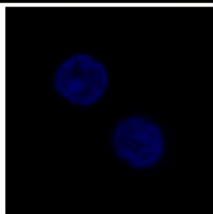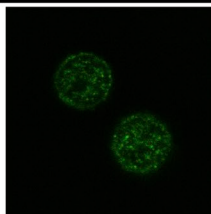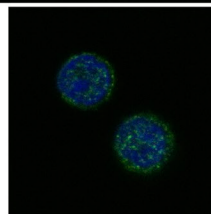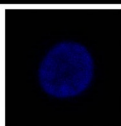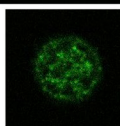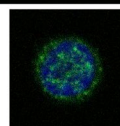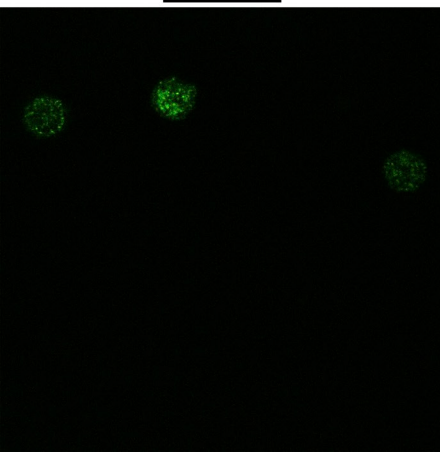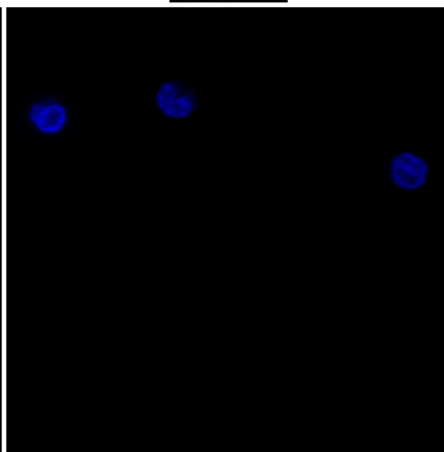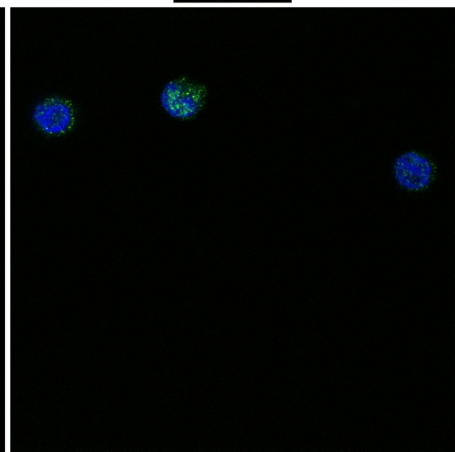

Supplement: SourceData F7 — is the source file for Fig. 7. [file JGP_202112865_SourceDataF7.pdf]

**S1B**

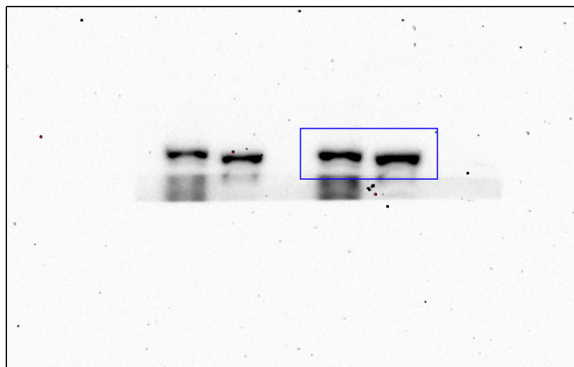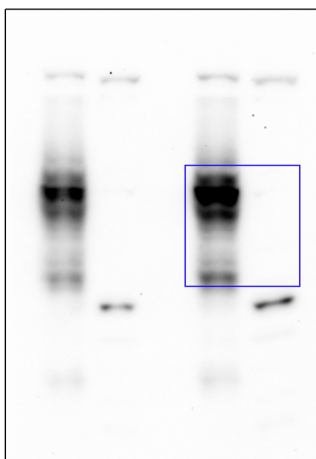

Supplement: SourceData FS1 — is the source file for Fig. S1. [file JGP_202112865_SourceDataFS1.pdf]
